# Human Tear Fluid Lipidome: From Composition to Function

**DOI:** 10.1371/journal.pone.0019553

**Published:** 2011-05-05

**Authors:** Antti H. Rantamäki, Tuulikki Seppänen-Laakso, Matej Oresic, Matti Jauhiainen, Juha M. Holopainen

**Affiliations:** 1 Helsinki Eye Lab, Department of Ophthalmology, University of Helsinki, Helsinki, Finland; 2 VTT Technical Research Centre of Finland, Espoo, Finland; 3 National Institute for Health and Welfare and FIMM Institute for Molecular Medicine Finland, Helsinki, Finland; George Mason University, United States of America

## Abstract

We have explored human aqueous tear fluid lipidome with an emphasis to identify the major lipids. We also address the physiological significance of the lipidome. The tears were analysed using thin layer chromatographic, enzymatic and mass spectrometric techniques. To emphasize the physiological aspect of the lipidome, we modelled the spreading of the non-polar tear fluid lipids at air-water interface in macroscopic scale with olive oil and egg yolk phosphatidylcholine. Based on enzymatic analysis the respective concentrations of choline-containing lipids, triglycerides, and cholesteryl esters were 48±14, 10±0, and 21±18 µM. Ultra performance liquid chromatography quadrupole time of flight mass spectrometry analysis showed that phosphatidylcholine and phosphatidylethanolamine were the two most common polar lipids comprising 88±6% of all identified lipids. Triglycerides were the only non-polar lipids detected in mass spectrometric analysis i.e. no cholesteryl or wax esters were identified. The spreading experiments show that the presence of polar lipids is an absolute necessity for a proper spreading of non-polar tear fluid lipids. We provide evidence that polar lipids are the most common lipid species. Furthermore, we provide a physiological rationale for the observed lipid composition. The results open insights into the functional role of lipids in the tear fluid and also aids in providing new means to understand and treat diseases of the ocular surface.

## Introduction

Tear fluid forms a tear film over the cornea and the conjunctiva of the eye (reviewed in [1] and [2]). It has several important functions regarding proper function and health of the ocular surface. It moistens the ocular surface, flushes contaminants out of the eye, protects the ocular surface against pathogens, lubricates the lid-cornea interface when blinking and sleeping, nourishes corneal epithelial cells, and improves optical properties by modifying refractive index of the cornea. The tear film has an ill-defined trilaminar and concentration-gradient-dependent structure. It consists roughly of an inner mucin-enriched mucous layer, a middle aqueous layer, and an outer lipid layer. The water-retaining and viscous nature of the mucous layer suggests that mucins increase the wetting properties and stability of the entire tear film. The aqueous layer contains tens or even hundreds of different proteins. Many proteins are involved in wound healing, inflammatory processes, as well as corneal protection from various pathogens [3]. Some of the proteins interact with the lipid layer and thus may have a biophysical function in stabilization and organization of the tear lipid layer. The aqueous layer also contains electrolytes and metabolites, but the tear metabolome has not been investigated in detail.

The lipid layer stabilizes the tear film by reducing overall surface free energy, i.e. surface tension, and by controlling water evaporation from the surface [1]. The lipid layer is in contact with the eyelid skin acting as a barrier to the aqueous layer [4]. Lipids also form a watertight seal when the lids are closed [1]. Part of this oily liquid is secreted from the meibomian glands [5]. Studies during past four decades have shown analysis-method-dependent variation in the composition of meibum [6]. Despite variations in the published compositions, sterol esters and wax esters seem to be the most abundant lipid species in the meibum. The lipid composition of tear fluid is far more complex than that of the meibum [7]. Although meibum lipids have been studied widely, comprehensive tear fluid lipidomic studies are lacking. Very few publications exist regarding the polar lipid composition of the tear fluid lipid layer. Ham and co-workers [8] reported the presence of sphingomyelins and phosphatidylcholines in the tear fluid. Recently also Saville and co-workers [9] discovered phospholipids both in meibum and tear fluid using electrospray ionization tandem mass spectrometry (ESI-MS/MS). Specifically, choline-containing lipids were targeted and the analysis revealed 11 sphingomyelins and 13 phosphatidylcholines in the tear fluid. Meibum had very similar lipid profile regarding the choline-containing lipids. Also the non-polar lipid composition of tear film seems to differ from that of meibum. In addition to triacylglycerols found in meibum, diacyl-, monoacylglycerols, and free glycerol were found in tear fluid [10]. Despite the fact that the tear samples in that study originated from rabbits, the results may well be extrapolated to humans. The source of the polar lipids, however, has not been shown. Butovich and co-workers proposed that the conjunctival and corneal epithelial cells may produce these lipids [7]. A plausible alternative would be that some specific cell types on ocular surface, similar to the type II alveolar epithelial cells in lungs, would specifically produce the polar lipids to the tear fluid. The similar properties and functions of the tear film and the lung surfactant support this suggestion [11]. These cells, if they exist, remain to be discovered.

Here we have analyzed in detail the lipid composition of the aqueous tear fluid. We show, in contrast to current view, that major proportion of the tear fluid lipids are polar phospholipids. Finally, we show that this type of composition is necessary for the function and stable spreading of the tear fluid lipid layer giving a physiological context to the present findings.

## Results

### Thin layer chromatography and enzymatic lipid assay

**Figure 1:**
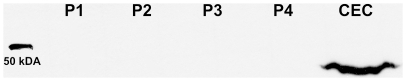
Western blot showing the negative cellular contamination of the tear samples. Four differing tear fluid pools P1–P4 were applied into the wells of SDS-PAGE gel. Equal amount of protein was loaded in each well. CEC, corneal epithelial cell lysate.

**Figure 2:**
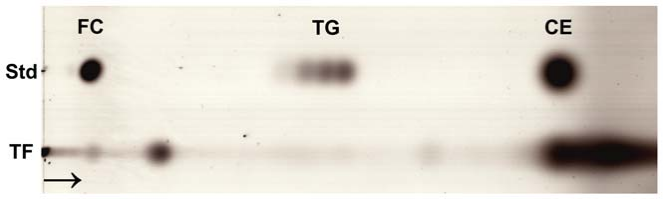
TLC of non-polar lipids. TF, tear fluid; Std, standard solution containing free cholesterol (FC), triglycerides (TG), and cholesteryl oleate (CE). The arrow shows the direction of the eluent flow.

Prior to detailed lipid analysis, we assessed the purity (i.e. cellular contamination) of the pooled samples, but we did not detect any bands that could have been identified as intracellular actin (Fig. 1.). Accordingly, no cellular contamination was expected. To get the first insight into the lipid status in tears we analyzed the aqueous tears by thin layer chromatographic (TLC) method. We performed two sets of chromatographic analysis, namely for polar and non-polar lipids. Polar lipids showed only very faint bands corresponding mainly to sphingomyelin (data not shown). In contrast, the analysis of non-polar lipids by TLC showed strong band migrating almost identically to that of cholesteryl esters (Fig. 2). Faint bands were also observed at the migration position of triglycerides and free cholesterol. To further confirm these findings we analyzed the lipid concentrations by enzymatic methods. The concentrations were 48±14 µM for choline-containing phospholipids, 10±0 µM for triglycerides, and 21±18 µM for total cholesterol. All the experiments were performed twice.

### Lipidomics

**Figure 3:**
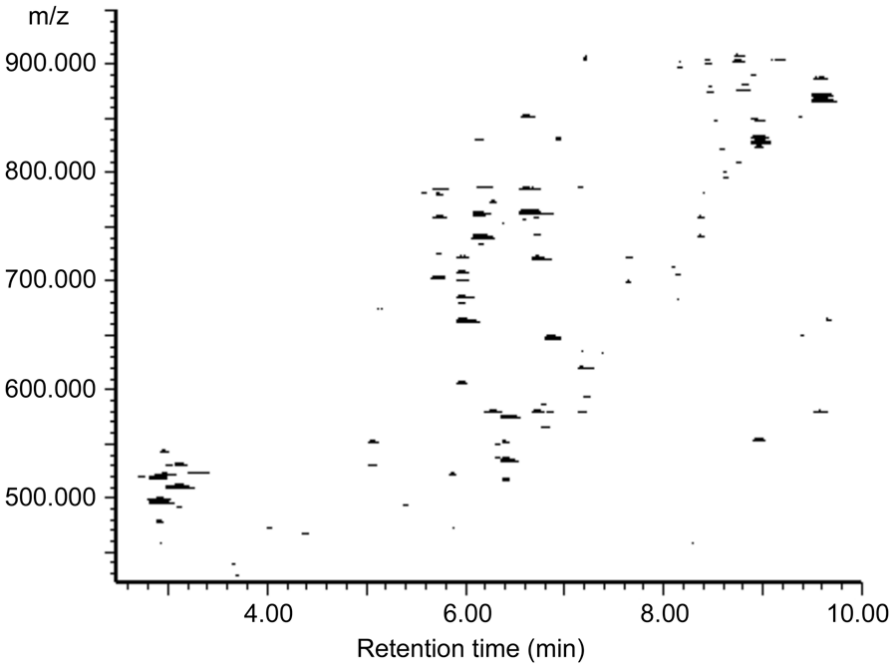
Two-dimensional map view of UPLC-MS data. The first dimension shows the retention times of the separated lipids and the second dimension reveals the detected lipid ions.

**Figure 4:**
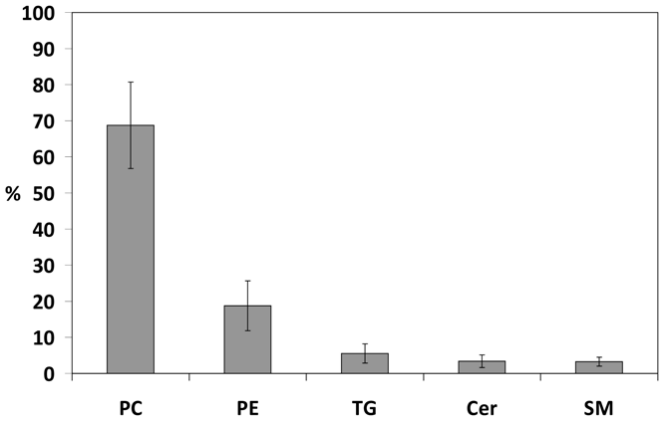
Molar percentage of the five most abundant lipid classes among the identified tear fluid lipids. The proportions are based on the UPLC-MS data. Cer, Ceramide; PC, phosphatidylcholine; PE, phosphatidylethanolamine; SM, sphingomyelin; TG, triglyceride.

Altogether we identified a total of 153 lipid species (Table S1) in 6 distinct lipid classes. Figure 3 illustrates the data obtained from ultra performance liquid chromatography mass spectrometry (UPLC-MS) experiments as a two-dimensional map view. Table 1 shows the concentrations for each lipid class, the total concentration of individual samples, and the mean values and standard deviations. Figure 4 shows average molar proportions of the five most abundant lipid classes among the identified lipids (phosphatidylserines are left out due to the negligible proportion). Polar lipids phosphatidylcholines (PC) and phosphatidylethanolamines (PE) formed ∼90 mol% of all identified lipids with respective proportions of ∼70% and ∼20%. The remaining proportion comprised of non-polar triglycerides (∼5%), polar sphingomyelins (SM, ∼3%), and ceramides (∼3%).

**Table 1:** Total concentrations of the identified tear fluid lipid classes.

class	S1 (µM)	S2 (µM)	mean (µM)	STD (µM)
PC	13.8	28.0	20.9	10.1
PE	5.3	5.2	5.2	0.1
TG	1.7	1.3	1.5	0.3
Cer	1.1	0.8	0.9	0.2
SM	0.9	0.9	0.9	0.1
PS	0.1	0.1	0.1	0.0
**TOTAL**	22.9	36.3	29.6	9.5

The concentrations are based on the UPLC-MS data obtained from the analysis of samples S1 and S2. Cer, Ceramide; PC, phosphatidylcholine; PE, phosphatidylethanolamine; PS, phosphatidyl-serine; SM, sphingomyelin; TG, triglyceride; TOTAL, total lipid concentration.

The ten most common lipids ((lysoPCs (16∶0), (18∶1), (18∶3), and (18∶0); plasmalogen PEs (34∶4) and (32∶6); PEs (36∶3) and (32∶3); PC (36∶3); and SM (d18∶1/16∶0)) formed together 66±8% of all of the identified lipids. The overall proportion of plasmalogens was 14±5% (designated with letter e in Table S1). The lipids containing unsaturated fatty acyl chains covered 54±11% of all identified lipids. We did not detect any free cholesterol or cholesteryl esters.

### Contact angle measurements

In order to elucidate the biology behind the lipidome data we considered the possibility that polar lipids and non-polar lipids could form a multilayered lipid phase [12], thus preventing evaporation of water from the tear fluid. It is clear that spreading of non-polar lipids at the air-water interface is troublesome because these lipids tend to form aggregates. In the blood this is solved by packing the non-polar lipids into the core compartment of globular lipoprotein particles. In the tear fluid this might, however, be somewhat problematic because lipid aggregates may contaminate the ocular surface. Accordingly, we hypothesized that in the tear fluid polar lipids are needed to form a platform for non-polar lipids to spread on. Although this is hypothesized in the literature [13], we have not been able to find such data from previous literature. Formation of this sandwich type planar layer would be stable and prevent water evaporation.

**Figure 5:**
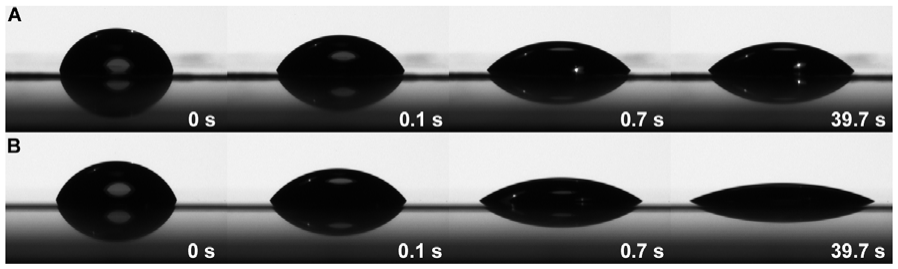
A photo sequence of olive oil spreading. (A) Blank mica and (B) eggPC monolayer on mica at π = 30 mN/m. Time points indicate the reading of the contact angles in second-scale. See Materials and Methods for more details.

The spreading of olive oil to blank and egg-yolk-phosphatidylcholine(eggPC)-coated mica surfaces is shown in Figure 5. The time points were chosen only for visualization purposes. On a blank mica surface at 0 s, the contact angle of a drop of olive oil was somewhat larger than on eggPC-coated surface. At 0.1 s the difference was already more evident with eggPC and blank mica surface providing contact angles of 57±2 and 71±6 degrees, respectively. At 0.7 s the spreading on the blank surface had stopped compared to the 39.7 s time point, where the contact angle had achieved its minimum values on both surfaces. On eggPC surface the spreading continued after 0.7 s and the minimum contact angles of 22±2 degrees at π = 20 mN/m and 24±1 degrees at π = 30 mN/m were significantly smaller compared to the minimum value of 45±1 degrees on the blank surface.

**Figure 6:**
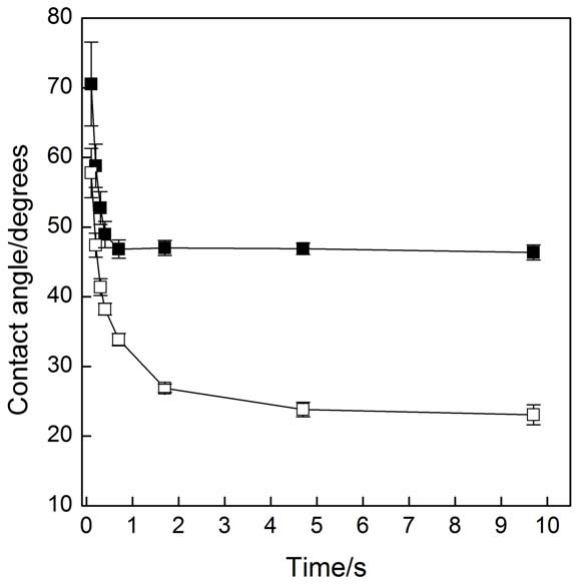
Spreading of olive oil: contact angle vs. time. (▪) Blank mica and (□) eggPC monolayer on mica at π = 30 mN/m. See Materials and Methods for more details.

Comparison of the spreading is also presented in Figure 6 as the change in contact angle as a function of time. A significant difference on the values of contact angles was observed already at the first time point and continued throughout the measurement (p<0.001, t-test). Values under 0.1 s are not shown because technical limitations of the recording technique. Due to the similarity of the spreading results for eggPC monolayers at π = 30 mN/m and π = 20 mN/m, the data for the latter is not shown.

## Discussion

In the present study we investigated the global lipidome of human tear fluid. We demonstrate that polar lipids formed the major proportion of all lipids, followed by cholesteryl esters and triglycerides. The most common phospholipids detected in mass spectrometric analysis were PC and PE, and these formed 88±6% of the identified lipids. Lysophospholipids formed a major portion of the lipids in MS analysis and suggest that phospholipase A2, a normal and abundant component of the tear fluid, is highly active [14]. The concentrations of choline-containing lipids obtained from enzymatic assays (48±14 µM) and MS experiments (22±10 µM, total of PC and SM in Table 1) are well in line with each other. It is worth noting, however, that the enzymatic lipid assays utilized in this study are developed for measuring lipid concentrations in blood plasma, which range from hundreds of micromoles to thousands of micromoles in litre. Therefore the concentrations obtained from these assays may be regarded only as indicative values. The concentrations of the individual pooled samples may also vary greatly. This is due to the fact that the samples are pooled randomly from several subjects and the individual differences of the subjects affect significantly the concentration of the final pooled sample. Pooled samples were analyzed, since obtaining adequate amounts of tear fluid from one subject without irritating the ocular surface is difficult.

TLC seems to be insensitive method for detecting phospholipids in tear fluid and were able to detect only faint band for SM in our samples. Wollensak and co-workers [15] managed to detect phospholipids in their tear samples using high performance TLC, but they were not able to detect phospholipids in all of their samples either. TLC shows an abundance of species in tear fluid of similar polarity to cholesteryl esters. The broad band identified as cholesteryl ester in Figure 2 most probably also contains the wax esters [15]. In line with TLC results, total cholesterol was also quantifiable with enzymatic lipid assay. The concentration of total cholesterol was approximated to equal the concentration of cholesteryl esters in tear fluid, since in meibum the cholesterol is mainly in the form of fatty acyl esters [16]. The total concentration of cholesterol was 40±27% of choline-containing lipid concentration. Triglycerides were the only non-polar lipids detected in our MS analysis. The amount of triglycerides relative to choline-containing lipids in MS analysis and enzymatic lipid assays were 8±5% and 22±6%, which are well in line with each other considering the differing analysis techniques. Also TLC showed faint bands for triglycerides.

Recently Chen and co-workers [16] identified several non-polar lipid classes in meibum using ESI-MS including cholesteryl esters, wax esters and triglycerides. In this present study, the only non-polar lipids we identified in tear fluid were triglycerides. Two factors may explain the absence of cholesteryl esters and wax esters in tear fluid MS spectra. First, tear fluid is extremely diluted compared to meibum. Secondly, the ionization efficiency of non-polar lipids in ESI is inefficient compared to polar phospholipids. Therefore the signal produced by the non-polar lipids may be too weak to be detected due to ion suppression in the presence of well-ionisable polar lipids, due to poor ionization of the non-polar lipids, and low overall concentration of the lipids. Another plausible reason why we did not detect cholesteryl esters in tear fluid is the fact that ionization of oxysterols in ESI is very poor [17], although cholesteryl esters as such are ionized [18]. Therefore the absence of cholesteryl esters may be due to the oxidation taking place on the ocular surface as the lipids in the tear fluid are a subject of oxidation due to the free-radical-producing UV radiation. Similarly, the inefficient ionization of free cholesterol may explain the absence of cholesterol-derived ions in MS spectra [18].

Intriguing finding was that 14±5% of all identified lipids were ether rather than ester lipids. Even though the exact structure of the ether lipids can not be defined based on the mass spectrometric analysis used, the ether lipids found are most probably plasmalogens as they are widely distributed in animals [19]. Also Saville and co-workers [9] reported plasmalogens in tear fluid. Plasmalogens are a peculiar class of glycerophospholipids with unique structure. Instead of an ester, plasmalogens have a vinyl-ether acyl chain linked to the *sn*-1 position of glycerol backbone. The *sn*-2 position has a “normal” oxyester bond. Plasmalogens are known to display an antioxidative function in lipid bilayers (reviewed in [20]). Morandat and co-workers have shown that plasmalogens also have an antioxidative function in lipid monolayers containing unsaturated phospholipids [21]. UV-radiation-generated reactive oxygen species react with some of the unsaturated phospholipids in tightly packed monolayers, and as a result peroxyl radicals are formed. The peroxyl radicals react further more rapidly with plasmalogens instead of unsaturated phospholipids and thus prevent the formation of substances that would lead into destruction of the lipid layer. Therefore plasmalogens impede the damage of UV-induced oxidative stress caused to unsaturated phospholipids. This view of the ether lipid function suits well also with the tear fluid lipid layer, as half of the lipids are unsaturated and the lipids are repeatedly exposured to UV light from the sun. The high abundance of ether lipids (14±5%) in tear fluid would also be relevant in the light of this theory, because the molar proportion of plasmalogens relative to the unsaturated lipids in the monolayer has to be greater than 25% in order to have an efficient antioxidative effect [21]. As nearly half of the lipids identified in tear fluid are unsaturated phospholipids, the molar proportion of plasmalogens to unsaturated phospholipids is 23±3%. In addition to the phospholipase A2 activity mentioned above, plasmalogen oxidation may also be another source of lysophospholipids as oxidized plasmalogens degrade into lysophospholipids and aldehydes [20].

Kulovesi and co-workers studied the three dimensional organization and behavior of an artificial tear fluid lipid layer comprising of 60 mol% of phospholipids, 20% free fatty acids, 10% cholesteryl esters and 10% triglycerides [12]. The lipidome obtained here coincides well with this model composition. Even though not observed in this study, the lipid layer most probably contains also free fatty acids because of the abundance of lysophospholipids. These results support the view demonstrated with the artificial model that the lipid layer is a highly organized layered structure i.e. phospholipid monolayer at the air-water interface provides a hydrophobic interface to where non-polar lipids are able to spread on. Here we emphasized the significance of polar/non-polar lipid interface in macroscopic scale with the non-polar olive oil spreading experiments. The contact angles (Fig. 5) on eggPC-monolayer-coated mica surface showed much lower values compared to the hydrophilic blank mica surface representing air-water interface. Both curves (Fig. 6) show similar shapes having a steep slope in early phase of the spreading, which then rapidly proceeds towards plateau. The curve illustrating spreading on eggPC surface achieves plateau at later time point compared to blank surface i.e. the spreading is more extensive on eggPC surface. Smaller error bars on eggPC surface at the early phase of spreading suggest more stable spreading. Therefore the spreading on eggPC surface proceeds more rapidly, is more stable, and more extensive finally achieving a smaller contact angle. On ocular surface this would mean that without the polar phospholipid interface the non-polar lipids would spread inadequately after a blink and also would form more unstable lipid layer. This would lead into faster tear film break-up and further increase in the evaporation rate of water from the tear fluid.

Taken together, our results are important in several ways. First of all, the lipidome of the tear fluid is very different than that of the meibomian gland secretions [6]. Phospholipids are very abundant in tear fluid and are most likely needed to provide a platform for the organization of the non-polar lipids at air-water interface [12]. Secondly, since the concentration of phospholipids seems to be negligble in the meibomian secretions [6] we need to consider the origin of the phospholipids. We suggest, in accordance with Butovich that they originate from the conjunctival and/or corneal epithelial cells [7], yet we have no solid experimental data to support this view. Thirdly, the high levels of plasmalogens is potentially important. It may well be that these lipids are mandatory for proper prevention of oxidative stress caused to polyunsaturated lipids. Finally, our data clearly demonstrate that non-polar lipids, when present at high amounts, are not able to spread normally to the air-water interface, and therefore phospholipids are an absolute necessity in aiding the spreading of the lipid layer. This sandwich-like organization is expected to prevent evaporation of water very efficiently.

## Materials and Methods

### Tear fluid collection

All experiments were performed in accordance with the guidelines of the Declaration of Helsinki and the Ethical Committee of the Helsinki-Uusimaa Hospital District approved this research. A written informed consent was obtained from each subject. Tear samples were collected from 30 healthy volunteers (age 20–55 years) from the lower conjunctival sac using 5 µl glass micropipets (Blaubrand Intramark, Brand GmbH, Wertheim, Germany). The collection was performed in multiple sessions under a biomicroscope using least possible irritation of the conjunctiva to minimize cellular contamination and sample dilution caused by reflex tearing. The samples were immediately cooled to +4°C, centrifuged at 13 000 rpm for 5 min to remove the possible contamination of cellular debris, and the collected supernatant was stored at −20°C until analyzed. Cellular contamination of the pooled samples was assessed by Western blotting using anti-actin rabbit polyclonal antibodies (Sigma-Aldrich, St. Louis, MO, USA). Corneal epithelial cell lysate was used as a positive control.

### Thin Layer Chromatography

Thin layer chromatography was performed for the lipids extracted from ∼80 µl of tear fluid by a method adapted from Folch and co-workers [22]. In short, first the proteins were precipitated from the tear fluid sample using chloroform-methanol solution. Then the lipids were extracted from the supernatant so that the whole composition of the solution (including the supernatant from the precipitation) was 8∶4∶3 chloroform/methanol/water (v/v). The upper phase was further extracted with chloroform/methanol/water (68∶14∶1) solution and the lower phases from both extractions were combined. The solvent was evaporated and the lipids were dissolved in chloroform. The sample was divided into two and applied to TLC Silica gel 60 glass plates (Merck KGaA, Darmstadt, Germany). TLC separations were run for polar and non-polar lipids. The eluent composition for polar lipid separation was chloroform/methanol/acetic acid/formic acid/water (70∶20∶12∶4∶2) and for non-polar lipids hexane/diethyl ether/acetic acid (80∶20∶1). After separation the plates were incubated in solution containing 3% (w/w) of copper sulfate and 8% (w/w) phosphoric acid and heat treated by raising the temperature during 30–45 min from ambient room temperature to 180°C. The charred lipids were detected from the plates by visual inspection and identified with the use of proper lipid standards. The extraction and separation of the lipids were performed twice.

### Enzymatic lipid assay

Total cholesterol (Roche Diagnostics GmbH, Mannheim, Germany), choline-containing phospholipids (DiaSys Diagnostic System GmbH, Holzheim, Germany) and triglycerides (Roche Diagnostics) were measured from tear fluid using fully enzymatic methods. The measurements were performed using Victor^2^ 1420 Multilabel Counter (Wallac Corp., Turku, Finland). The respective assays for each class were performed twice.

### Lipidomics

The lipidomics platform based on UPLC connected to ESI quadruple time-of-flight (Q-TOF) mass spectrometry was utilized. The applied platform [23] affords extensive screening of multiple lipid classes from total lipid extracts within a single sample run. Since a diverse range of lipids such as phospholipids and non-polar lipids were of interest, the experiments were performed in positive ion mode.

With UPLC, separation of the lipids can be achieved in 10 minutes without significant loss in sensitivity, covering major monoacylglycerols, diacylglycerols, triglycerides and phospholipids, lysophospholipids, sphingolipids, and cholesteryl esters. Similar method has been utilized in multiple clinical and preclinical studies [24]–[27].

### Lipid extraction

Tear samples collected from the subjects were pooled and formed two separate samples for lipidomic analysis. The pooled tear samples (2×75 µl) were spiked in 0.5 ml Eppendorf tubes with 20 µl of a standard mixture containing 10 different lipid class compounds (lysoPC, PC, PE, phosphatidylserine, phosphatidic acid, phosphatidylglycerol, ceramide, monoacylglycerol, diacylglycerol, and triglyceride as their heptadecanoic deriavatives) at the levels of 0.18–0.22 µg. Lipids were extracted with 100 µl of chloroform∶methanol (2∶1) by vortexing for 2 min at room temperature. After 1 hour standing the samples were centrifuged at 10 000 rpm for 3 minutes and the lower organic solvent layers were separated into glass vials containing another lipid standard mixture (labelled lysoPC, PC and triglyceride) standards at a concentration level of 0.1 µg). To concentrate the samples, they were combined, evaporated into dryness, and dissolved into 20 µl of chloroform∶methanol (2∶1). The ratios of the unlabelled and labelled standards were used to control the extraction procedure. In the sample set, non-extracted and extracted standard samples were also included to show the validity of the extraction.

### UPLC-QTOF-MS analysis

The tear lipid extract was analysed on a Waters Q-TOF Premier mass spectrometer combined with an Acquity Ultra Performance LC (UPLC). The column (at 50°C) was an Acquity UPLC™ BEH C18 2.1×100 mm with 1.7 µm particles. The solvent system included A. ultrapure water (1% 1 M NH_4_Ac, 0.1% HCOOH) and B. LC/MS grade acetonitrile/isopropanol (1∶1 (v/v), 1% 1 M NH_4_Ac, 0.1% HCOOH). The gradient started from 65% A/35% B, reached 80% B in 2 min and 100% B in 7 min, and remained there for the next 7 min. There was a 4 min re-equilibration step before next run. The flow rate was 0.4 ml/min and the injected sample volume 2.0 µl (Acquity Sample Organizer). Reserpine was used as the lock spray reference compound. The lipid profiling was carried out using ESI+ mode and the data was collected at mass range of m/z 300–1200 with scan duration of 0.2 sec. The data was processed by using MZmine 2 software [28] and the lipid identification was based on an internal spectral library [29].

### Contact Angle Measurements

The samples for the contact angle measurements were prepared by the Langmuir-Blodgett technique, i.e. transferring a lipid monolayer from air-water interface to a solid support. The freshly cleaved mica sheets (Agar Scientific Ltd, Stansted, UK) were submerged vertically through the air-water interface of the Langmuir device (model Minitrough 2000, KSV Instruments Ltd., Helsinki, Finland). Egg yolk phosphatidylcholine (eggPC, ∼99%, Sigma-Aldrich, St. Louis, MO, USA) dissolved in chloroform (HPLC grade, Sigma-Aldrich, St. Louis, MO, USA) was spread in small aliquots onto the interface. Before the transfer the monolayer was compressed and relaxed twice to the transfer surface pressure. Two different transfer surface pressures, π, were used, 20 and 30 mN/m. The transfer was done at controlled constant surface pressure by elevating the mica substrate through the air-water interface at the rate of 2 mm/min. The transfer ratios from air-water interface to the mica solid support were approximately 1∶1. Representing a hydrophilic water interface, blank freshly cleaved mica sheets were used as a reference material in the contact angle measurements.

The contact angles were measured with a video camera based and computer controlled device (CAM 200, KSV Instruments Ltd., Helsinki, Finland). The contact angles of the drops were determined with the CAM200 software by fitting method based on the Young and Laplace equation. The liquid used for contact angle measurements was commercially available extra virgin olive oil containing 10% (w/w) of saturated, 80% of monounsaturated and 10% of polyunsaturated fatty acids. Olive oil is mainly composed of triglycerides and small amounts of free fatty acids. In this study it was used as a simple model representing the non-polar lipids of the tear fluid.

An aliquot of olive oil was applied to the mica surface with a threaded plunger syringe (Hamilton Co., Reno, USA). The volume of the drop hanging on the needle was increased slowly until the drop was detached from the tip. The camera started recording automatically when the drop detached the tip of the needle first taking 21 pictures every 33 ms and then 39 pictures every second. After recording, the contact angles of the drops were defined in 0.1, 0.2, 0.3, 0.4, 0.7, 1.7, 4.7, 9.7 and 39.7 second intervals. At least six contact angle values were measured for each time point using two duplicate samples (3 drops per sample). Contact angles are expressed as mean ± standard deviation. Student's t-test was used for comparison between the contact angles obtained on blank mica surface and eggPC-coated mica surface. A p-value<0.05 was considered significant.

## Supporting Information

Table S1 **Lipids identified in UPLC-MS analysis.** RT, retention time; S1, sample 1; S2, sample 2.
(DOC)
