# Supplementary material for: Human Tear Fluid Lipidome: From Composition to Function
Source: PLoS One. 2011 May 5;6(5):e19553. doi: 10.1371/journal.pone.0019553 (PMC3088682; doi:10.1371/journal.pone.0019553)
Supplement: Table S1 — Lipids identified in UPLC-MS analysis. RT, retention time; S1, sample 1; S2, sample 2. (DOC) [file pone.0019553.s001.doc]

| **Id** | **m/z** | **RT (s)** | **Name** | **Id type** | **S1 (µM)** | **S2 (µM)** |
| --- | --- | --- | --- | --- | --- | --- |
| 554 | 496.3406 | 175.2 | LysoPC(16:0) | MS | 6.6571 | 15.5609 |
| 410 | 522.3538 | 178.3 | LysoPC(18:1) | MS | 1.4747 | 2.9852 |
| 29 | 685.4354 | 357.1 | PE(34:4e) | MS | 1.2078 | 0.9987 |
| 592 | 518.3208 | 176.2 | LysoPC(18:3) | MS | 1.1150 | 1.6909 |
| 358 | 524.3730 | 199.0 | LysoPC(18:0) | MS | 0.7911 | 2.4141 |
| 62 | 742.5379 | 403.7 | PE(36:3) | MS | 0.5809 | 0.5417 |
| 467 | 666.4578 | 357.4 | PE(32:6e) | MS | 0.5593 | 0.5133 |
| 407 | 784.5846 | 396.3 | PC(36:3) | MS | 0.5370 | 0.6132 |
| 114 | 686.4398 | 357.1 | PE(32:3) | MS | 0.5061 | 0.4458 |
| 121 | 703.5771 | 342.8 | SM(d18:1/16:0) | MS | 0.4145 | 0.3727 |
| 106 | 721.5589 | 402.7 | PC(32:0e) | MS | 0.4095 | 0.5002 |
| 194 | 536.5334 | 384.4 | Cer(d18:1/16:1) | MS | 0.4041 | 0.3379 |
| 180 | 758.5683 | 342.8 | PC(34:2) | MS-MS | 0.3839 | 0.4122 |
| 344 | 516.5147 | 384.1 | Cer(d18:1/17:0) | MS | 0.3788 | 0.3013 |
| 297 | 722.5662 | 403.4 | PE(36:6e) | MS | 0.3729 | 0.3717 |
| 509 | 902.8210 | 524.8 | TG(54:3) | MS | 0.3189 | 0.1395 |
| 449 | 762.5892 | 368.1 | PC(34:0) | MS | 0.3099 | 0.3373 |
| 582 | 722.5103 | 357.1 | PE(36:6e) | MS | 0.2965 | 0.2374 |
| 457 | 544.3385 | 178.6 | LysoPC(20:4) | MS | 0.2902 | 0.6227 |
| 475 | 742.6160 | 367.8 | PC(34:3e) | MS | 0.2188 | 0.3100 |
| 259 | 773.5329 | 376.2 | PE(38:2) | MS | 0.2161 | 0.2371 |
| 302 | 520.3401 | 164.3 | LysoPC(18:2) | MS | 0.1951 | 0.4287 |
| 504 | 478.3302 | 175.6 | LysoPE(18:2) | MS | 0.1905 | 0.4299 |
| 21 | 701.4133 | 355.5 | PE(34:3e) | MS | 0.1879 | 0.1269 |
| 46 | 709.5169 | 357.1 | PE(34:5) | MS | 0.1818 | 0.1458 |
| 254 | 580.5395 | 429.8 | Cer(d18:1/19:0) | MS | 0.1675 | 0.0663 |
| 400 | 760.5864 | 368.4 | PC(34:1) | MS | 0.1331 | 0.1443 |
| 234 | 764.5992 | 368.1 | PE(40:6e) | MS | 0.1089 | 0.1144 |
| 210 | 774.5368 | 376.2 | PE(40:8e) | MS | 0.1068 | 0.1150 |
| 565 | 850.7761 | 535.1 | TG(16:0/18:1/16:0) | MS-MS | 0.1064 | 0.0892 |
| 406 | 784.5867 | 343.7 | PC(36:3) | MS-MS | 0.1034 | 0.1147 |
| 393 | 786.6039 | 372.2 | PC(36:2) | MS-MS | 0.0983 | 0.1260 |
| 14 | 725.5565 | 342.8 | SM(d18:1/18:3) | MS | 0.0938 | 0.0994 |
| 354 | 759.6433 | 403.1 | SM(d18:1/20:0) | MS | 0.0863 | 0.0831 |
| 303 | 520.3329 | 175.6 | LysoPC(18:2) | MS | 0.0805 | 0.1625 |
| 188 | 675.5427 | 308.0 | SM(d18:1/14:0) | MS-MS | 0.0795 | 0.0665 |
| 70 | 795.5140 | 376.2 | PE(40:5) | MS | 0.0793 | 0.0759 |
| 766 | 764.5562 | 411.2 | PE(38:6) | MS | 0.0787 | 0.1017 |
| 251 | 830.5815 | 368.4 | PC(40:8) | MS | 0.0784 | 0.0854 |
| 328 | 566.5742 | 407.5 | Cer(d18:1/18:0) | MS | 0.0770 | 0.0529 |
| 357 | 524.3645 | 177.7 | LysoPC(18:0) | MS | 0.0757 | 0.2347 |
| 63 | 908.7830 | 524.8 | TG(55:7) | MS | 0.0737 | 0.0382 |
| 195 | 876.8077 | 527.9 | TG(52:2) | MS | 0.0727 | 0.0349 |
| 453 | 848.7763 | 512.9 | TG(14:0/18:1/18:1)+TG(16:0/16:1/18:1) | MS-MS | 0.0659 | 0.0665 |
| 628 | 780.5528 | 343.4 | PC(36:5) | MS | 0.0638 | 0.0634 |
| 150 | 546.3556 | 199.3 | LysoPC(20:3) | MS | 0.0638 | 0.1619 |
| 443 | 482.3275 | 164.3 | LysoPE(18:0) | MS | 0.0636 | 0.1056 |
| 555 | 760.6406 | 403.1 | PE(38:1e) | MS | 0.0635 | 0.0536 |
| 412 | 815.7029 | 452.0 | SM(d18:1/24:0) | MS-MS | 0.0614 | 0.0589 |
| 325 | 824.7779 | 537.0 | TG(48:0) | MS | 0.0596 | 0.0572 |
| 321 | 726.5605 | 342.4 | PE(36:4e) | MS | 0.0581 | 0.0557 |
| 561 | 494.3249 | 159.2 | LysoPC(16:1) | MS | 0.0570 | 0.1450 |
| 501 | 731.6091 | 375.9 | SM(d18:1/18:0) | MS-MS | 0.0564 | 0.0565 |
| 560 | 930.8590 | 545.2 | TG(18:1/18:1/20:1) | MS-MS | 0.0562 | 0.0179 |
| 269 | 904.8303 | 524.8 | TG(54:2) | MS | 0.0546 | 0.0250 |
| 543 | 786.5943 | 397.2 | PC(36:2) | MS | 0.0541 | 0.0622 |
| 282 | 744.5394 | 403.7 | PE(36:2) | MS | 0.0537 | 0.0595 |
| 167 | 813.6908 | 423.7 | SM(d18:1/24:1) | MS-MS | 0.0523 | 0.0497 |
| 192 | 768.6388 | 394.5 | PE(40:4e) | MS | 0.0498 | 0.0520 |
| 123 | 874.7906 | 507.8 | TG(52:3) | MS | 0.0492 | 0.0278 |
| 267 | 534.3406 | 187.5 | LysoPS(19:3) | MS | 0.0488 | 0.0411 |
| 454 | 768.5350 | 357.4 | PE(38:4) | MS | 0.0479 | 0.0431 |
| 540 | 732.6097 | 375.6 | PE(36:1e) | MS | 0.0466 | 0.0372 |
| 432 | 848.7143 | 537.0 | TG(50:2) | MS | 0.0457 | 0.0489 |
| 364 | 904.8381 | 550.1 | TG(54:2) | MS | 0.0445 | 0.0198 |
| 258 | 738.5906 | 369.0 | PE(38:5e) | MS | 0.0441 | 0.0351 |
| 196 | 750.5343 | 355.5 | PE(38:6e) | MS | 0.0422 | 0.0360 |
| 181 | 752.5670 | 383.5 | PE(38:5e) | MS | 0.0422 | 0.0356 |
| 156 | 900.8083 | 505.7 | TG(54:4) | MS | 0.0420 | 0.0282 |
| 144 | 782.5712 | 335.2 | PC(36:4) | MS-MS | 0.0410 | 0.0575 |
| 578 | 796.7430 | 516.0 | TG(46:0) | MS | 0.0407 | 0.0426 |
| 190 | 701.5569 | 309.9 | SM(d18:1/16:1) | MS-MS | 0.0384 | 0.0298 |
| 456 | 538.5390 | 379.0 | Cer(d18:1/16:0) | MS | 0.0381 | 0.0255 |
| 821 | 722.5592 | 347.1 | PE(36:6e) | MS | 0.0369 | 0.0635 |
| 294 | 468.3132 | 153.7 | LysoPC(14:0) | MS | 0.0354 | 0.0765 |
| 245 | 781.6264 | 403.1 | SM(d18:1/22:3) | MS | 0.0350 | 0.0286 |
| 142 | 480.3210 | 181.4 | LysoPE(18:1) | MS | 0.0336 | 0.0687 |
| 365 | 734.5683 | 368.7 | PC(32:0) | MS | 0.0317 | 0.0396 |
| 418 | 898.7869 | 490.7 | TG(54:5) | MS | 0.0313 | 0.0280 |
| 472 | 822.7617 | 516.0 | TG(48:1) | MS | 0.0309 | 0.0437 |
| 201 | 810.7584 | 525.4 | TG(47:0) | MS | 0.0302 | 0.0309 |
| 69 | 687.4421 | 359.5 | PC(30:3e) | MS | 0.0302 | 0.0315 |
| 149 | 568.3568 | 156.4 | LysoPC(22:6) | MS | 0.0298 | 0.0215 |
| 408 | 890.8200 | 535.4 | TG(54:9) | MS | 0.0272 | 0.0113 |
| 862 | 482.3279 | 203.3 | LysoPC(16:0e) | MS | 0.0272 | 0.0383 |
| 889 | 550.3873 | 201.5 | LysoPS(20:2) | MS | 0.0261 | 0.0449 |
| 888 | 542.3260 | 164.3 | LysoPC(20:5) | MS | 0.0259 | 0.0633 |
| 553 | 852.8074 | 561.6 | TG(16:0/16:0/18:0) | MS-MS | 0.0256 | 0.0245 |
| 836 | 755.6255 | 383.8 | SM(d18:1/20:2) | MS | 0.0254 | 0.0250 |
| 101 | 782.5678 | 368.1 | PC(36:4) | MS | 0.0244 | 0.0252 |
| 556 | 754.6271 | 383.5 | PC(36:4e) | MS | 0.0232 | 0.0225 |
| 757 | 723.5232 | 357.4 | PC(32:5) | MS | 0.0228 | 0.0253 |
| 417 | 782.7268 | 503.2 | TG(45:0) | MS | 0.0226 | 0.0225 |
| 126 | 880.7493 | 508.1 | TG(52:0) | MS | 0.0216 | 0.0152 |
| 584 | 882.7650 | 528.8 | TG(53:6) | MS | 0.0213 | 0.0145 |
| 1 | 808.5864 | 334.3 | PC(38:5) | MS-MS | 0.0209 | 0.0288 |
| 867 | 454.3004 | 177.7 | LysoPE(16:0) | MS | 0.0203 | 0.0323 |
| 263 | 906.7604 | 506.6 | TG(54:1) | MS | 0.0203 | 0.0109 |
| 323 | 806.5661 | 342.8 | PC(38:6) | MS | 0.0201 | 0.0212 |
| 154 | 740.6884 | 484.4 | TG(42:0) | MS | 0.0200 | 0.0161 |
| 208 | 838.7969 | 548.5 | TG(49:0) | MS-MS | 0.0195 | 0.0201 |
| 567 | 768.7161 | 499.5 | TG(44:0) | MS | 0.0192 | 0.0152 |
| 689 | 546.3432 | 178.3 | LysoPC(20:3) | MS | 0.0186 | 0.0353 |
| 568 | 794.7294 | 497.1 | TG(46:1) | MS | 0.0180 | 0.0292 |
| 708 | 506.3662 | 199.9 | LysoPC(18:2e) | MS | 0.0180 | 0.0542 |
| 148 | 808.7433 | 506.0 | TG(47:1) | MS | 0.0175 | 0.0240 |
| 177 | 880.8422 | 587.8 | TG(52:0) | MS | 0.0174 | 0.0175 |
| 392 | 786.5986 | 343.4 | PC(36:2) | MS | 0.0173 | 0.0173 |
| 143 | 782.5751 | 317.8 | PC(36:4) | MS | 0.0172 | 0.0165 |
| 356 | 928.8451 | 524.2 | TG(56:4) | MS-MS | 0.0172 | 0.0074 |
| 653 | 544.3425 | 161.4 | LysoPC(20:4) | MS | 0.0164 | 0.0224 |
| 9 | 810.6024 | 364.0 | PC(38:4) | MS-MS | 0.0163 | 0.0185 |
| 430 | 906.8539 | 581.7 | TG(54:1) | MS | 0.0162 | 0.0095 |
| 448 | 788.6167 | 395.4 | PC(36:1) | MS | 0.0161 | 0.0177 |
| 693 | 508.3437 | 168.9 | LysoPE(20:1) | MS | 0.0159 | 0.0319 |
| 702 | 504.2998 | 164.6 | LysoPE(20:3) | MS | 0.0158 | 0.0188 |
| 80 | 836.7759 | 524.2 | TG(49:1) | MS-MS | 0.0158 | 0.0232 |
| 250 | 800.5567 | 396.6 | PC(38:2e) | MS | 0.0158 | 0.0193 |
| 2 | 808.5857 | 372.5 | PC(38:5) | MS | 0.0154 | 0.0193 |
| 225 | 820.7455 | 497.5 | TG(48:2) | MS-MS | 0.0153 | 0.0273 |
| 688 | 546.3622 | 168.9 | LysoPC(20:3) | MS | 0.0151 | 0.0331 |
| 184 | 904.7437 | 488.8 | TG(54:2) | MS | 0.0145 | 0.0147 |
| 861 | 482.3668 | 186.0 | LysoPC(16:0e) | MS | 0.0142 | 0.0214 |
| 375 | 894.8457 | 604.5 | TG(53:0) | MS | 0.0141 | 0.0130 |
| 342 | 834.7616 | 504.1 | TG(49:2) | MS | 0.0138 | 0.0183 |
| 447 | 788.6092 | 371.9 | PC(36:1) | MS | 0.0131 | 0.0156 |
| 322 | 726.5880 | 353.4 | PC(34:4e) | MS | 0.0129 | 0.0126 |
| 186 | 796.5141 | 375.9 | PC(38:4e) | MS | 0.0126 | 0.0114 |
| 507 | 896.7775 | 476.0 | TG(54:6) | MS | 0.0126 | 0.0144 |
| 445 | 888.8145 | 515.1 | TG(53:3) | MS | 0.0125 | 0.0086 |
| 844 | 878.8255 | 555.0 | TG(52:1) | MS | 0.0125 | 0.0112 |
| 174 | 724.5240 | 356.2 | PC(32:5) | MS | 0.0124 | 0.0142 |
| 550 | 788.6729 | 428.6 | TG(46:4) | MS | 0.0123 | 0.0158 |
| 308 | 864.8090 | 540.7 | TG(51:1) | MS | 0.0115 | 0.0112 |
| 224 | 878.8227 | 527.9 | TG(52:1) | MS | 0.0114 | 0.0070 |
| 459 | 832.5941 | 367.4 | PC(40:7) | MS | 0.0112 | 0.0121 |
| 317 | 816.7094 | 453.2 | TG(48:4) | MS | 0.0112 | 0.0152 |
| 381 | 910.8000 | 550.4 | TG(55:6) | MS | 0.0112 | 0.0073 |
| 124 | 874.7859 | 572.0 | TG(52:3) | MS | 0.0111 | 0.0101 |
| 714 | 806.5683 | 323.3 | PC(38:6) | MS-MS | 0.0111 | 0.0151 |
| 78 | 908.8658 | 621.3 | TG(54:0) | MS | 0.0109 | 0.0135 |
| 429 | 906.8468 | 550.4 | TG(54:1) | MS | 0.0106 | 0.0064 |
| 525 | 792.7100 | 483.7 | TG(46:2) | MS-MS | 0.0105 | 0.0140 |
| 593 | 854.7328 | 511.4 | TG(51:6) | MS | 0.0104 | 0.0096 |
| 241 | 902.7337 | 476.3 | TG(54:3) | MS | 0.0103 | 0.0115 |
| 856 | 766.7038 | 482.5 | TG(44:1) | MS-MS | 0.0100 | 0.0111 |
| 898 | 872.7744 | 492.5 | TG(52:4) | MS | 0.0099 | 0.0136 |
| 906 | 516.3123 | 159.8 | LysoPC(18:4) | MS | 0.0096 | 0.0216 |
| 829 | 814.6988 | 425.6 | TG(48:5) | MS | 0.0094 | 0.0110 |
| 915 | 806.7291 | 488.2 | TG(47:2) | MS | 0.0079 | 0.0168 |
| 905 | 846.7610 | 497.1 | TG(50:3) | MS-MS | 0.0072 | 0.0130 |
| 679 | 818.7367 | 482.5 | TG(48:3) | MS-MS | 0.0067 | 0.0108 |
| 795 | 828.6990 | 410.6 | TG(49:5) | MS | 0.0048 | 0.0208 |
